# Common variation contributes to the genetic architecture of social communication traits

**DOI:** 10.1186/2040-2392-4-34

**Published:** 2013-09-18

**Authors:** Beate St Pourcain, Andrew J O Whitehouse, Wei Q Ang, Nicole M Warrington, Joseph T Glessner, Kai Wang, Nicholas J Timpson, David M Evans, John P Kemp, Susan M Ring, Wendy L McArdle, Jean Golding, Hakon Hakonarson, Craig E Pennell, George Davey Smith

**Affiliations:** 1 MRC Integrative Epidemiology Unit, University of Bristol, Oakfield House, Oakfield Grove, Bristol BS8 2BN, UK; 2 School of Oral and Dental Sciences, University of Bristol, Bristol, UK; 3 School of Experimental Psychology, University of Bristol, Bristol, UK; 4 Telethon Institute for Child Health Research, Centre for Child Health Research, University of Western Australia, Perth, Australia; 5 School of Psychology, University of Western Australia, Perth, Australia; 6 School of Women’s and Infants’ Health, University of Western Australia, Perth, Australia; 7 Children’s Hospital of Philadelphia, Philadelphia, PA, USA; 8 Zilkha Neurogenetic Institute & Department of Psychiatry, University of Southern California, Los Angeles, CA, USA; 9 School of Social and Community Medicine, University of Bristol, Oakfield House, Oakfield Grove, Bristol BS8 2BN, UK

**Keywords:** ALSPAC, RAINE, Autistic trait, GWAS, Social communication, Association

## Abstract

**Background:**

Social communication difficulties represent an autistic trait that is highly heritable and persistent during the course of development. However, little is known about the underlying genetic architecture of this phenotype.

**Methods:**

We performed a genome-wide association study on parent-reported social communication problems using items of the children’s communication checklist (age 10 to 11 years) studying single and/or joint marker effects. Analyses were conducted in a large UK population-based birth cohort (Avon Longitudinal Study of Parents and their Children, ALSPAC, N = 5,584) and followed-up within a sample of children with comparable measures from Western Australia (RAINE, N = 1364).

**Results:**

Two of our seven independent top signals (*P-*discovery <1.0E-05) were replicated (0.009 <*P-*replication ≤0.02) within RAINE and suggested evidence for association at 6p22.1 (rs9257616, meta-*P* = 2.5E-07) and 14q22.1 (rs2352908, meta-*P* = 1.1E-06). The signal at 6p22.1 was identified within the olfactory receptor gene cluster within the broader major histocompatibility complex (MHC) region. The strongest candidate locus within this genomic area was *TRIM27*. This gene encodes an ubiquitin E3 ligase, which is an interaction partner of methyl-CpG-binding domain (MBD) proteins, such as MBD3 and MBD4, and rare protein-coding mutations within *MBD3* and *MBD4* have been linked to autism. The signal at 14q22.1 was found within a gene-poor region.

Single-variant findings were complemented by estimations of the narrow-sense heritability in ALSPAC suggesting that approximately a fifth of the phenotypic variance in social communication traits is accounted for by joint additive effects of genotyped single nucleotide polymorphisms throughout the genome (h^2^(SE) = 0.18(0.066), *P* = 0.0027).

**Conclusion:**

Overall, our study provides both joint and single-SNP-based evidence for the contribution of common polymorphisms to variation in social communication phenotypes.

## Background

Autism spectrum disorders (ASDs) demarcate the extreme end of a continuum of behavioural difficulties [1], characterised by impairments of social interaction and communication as well as highly restricted interests and/or stereotyped repetitive behaviours [2]. The subthreshold end of this continuum is embodied by ASD-related but milder and non-psychopathological phenotypes, which are, as ASD, highly heritable (h^2^ = 0.36 to 0.87 [3-9]) and highly persistent [10,11] throughout the course of development.

Twin studies have reported no difference in heritability estimates of autistic symptomatology between the extremes of the distribution and normal variation [7,8], suggesting that clinical ASD and autistic-like traits in the general population may be etiologically linked. It is therefore possible that some variants influencing the expression of autistic traits might indeed represent underlying ASD quantitative trait loci (QTL). This assumption is supported by studies showing that common genetic variation at 5p14 [12] carries not only risk for ASD but is also associated with the expression of social communication spectrum phenotypes in the general population [13]. Candidate gene association studies identified furthermore *CYP11B1* and *NTRK1* as possible candidate loci, which may contribute to both risk of autism and the expression of autistic traits [14]. Twin studies, however, also suggested that there is heterogeneity among the three components of the autistic triad, and that social communication spectrum phenotypes, which are heritable traits [6,15], are potentially aetiologically distinct from other autistic behavioural domains [15,16].

While there are multiple efforts to investigate quantitative traits within autism samples both through linkage [17-20] and association designs [21], there is currently little known about the nature of genetic variants affecting autistic traits in the general population. The largest genome-wide effort to date has been conducted by Ronald and colleagues, using a DNA pooling approach in high- versus low-scoring individuals with respect to social and non-social autistic-like traits [22]. Although one SNP was replicated within an independent sample, the signal did not reach genome-wide significance. This might be related to some (expected) power loss because of inaccurate calls during the DNA pooling stage. Given the possibility of genetic links between the extreme and the subthreshold end of the autistic spectrum, however, a powerful genome-wide analysis of autistic traits analysed dimensionally in the general population may provide an opportunity to gain insights into the common genetic architecture of the autistic dimension. This is important, as common genetic variation identified by genome-wide association studies (GWAS) in ASD samples [12,23-27] has so far been either not replicated in more than one study [28], or did not reach evidence for genome-wide significance. Analyses of joint SNP effects suggested furthermore that the effect of common variation on risk for ASD is modest [24], highlighting the importance of study power, while other studies suggested that the lack of replication might be partially due to the underlying genetic heterogeneity of ASD, which in turn might be linked to different ASD subtypes [21]. In this context, it seems surprising that the effect of a common ASD GWAS signal at 5p14 [12] could be detected within a large population-based cohort investigating a continuum of broader ASD-related traits [13]. However, cohort designs encompass considerable advantages that can assist in the discovery of common genetic variation: cohort samples are in general large and thus highly powerful study populations, they are robust towards the influence of rare mutations of large effects and trait information can be uniformly assessed with validated instruments across an entire continuum, including both the sub-threshold end and the affected extreme.

Our study aimed to identify common variation in social communication spectrum phenotypes in the general population using GWAS. Association signals were discovered within a large UK population-based birth cohort, the Avon Longitudinal Study of Parents and their Children (ALSPAC) for which the continuity of ASD-related traits has been demonstrated [29,30], and followed-up in the Western Australian Pregnancy Cohort (RAINE) Study. Here we report support for single SNP association at 6p22.1 and 14q22.1 based on replication in independent samples.

## Methods

### Study populations

*ALSPAC* is a population-based longitudinal pregnancy-ascertained birth cohort in the Bristol area of the UK, with an estimated date of birth between 1 April 1991 and 31 December 1992 [31]. The initial cohort included 14,541 pregnancies and additional children eligible using the original enrolment definition were recruited up to the age of 18 years, increasing the total number of pregnancies to 15,247. The cohort is representative of the general population (approximately 96% white mothers, based on self-report). Information on the children from these pregnancies is available from questionnaires, clinical assessments, linkage to health and administrative records as well as biological samples. Ethical approval was obtained from the ALSPAC Law and Ethics Committee (IRB00003312) and the Local Research Ethics Committees, and written informed consent was provided by all parents.

*RAINE* is a longitudinal investigation of 2,900 pregnant women and their offspring consecutively recruited from maternity units between 1989 and 1991 [32]. The inclusion criteria were (i) English language skills sufficient to understand the study demands, (ii) an expectation to deliver at King Edward Memorial Hospital (KEMH), and (iii) an intention to remain in Western Australia to enable future follow-up of their child. Ninety percent of eligible women agreed to participate in the study. From the original cohort, 2,868 children have been followed over two decades. Participant recruitment and all follow-ups of their families were approved by the Human Ethics Committee at KEMH and/or Princess Margaret Hospital for Children in Perth. The RAINE sample is representative of the larger Australian population (88% Caucasian). DNA samples have been collected using standardised procedures at 14 or 16 years of age. Only those children with both biological parents of White European origin, based on self-report, were included in the current analyses.

### Phenotype selection

Social communication difficulties in ALSPAC children were measured at the age of 10 years based on mother-report using the 38-item pragmatic composite score of the Children’s Communication Checklist (CCC) [33]. Moderate to high levels of heritability (0.56 <h^2^<1) have been demonstrated for all CCC subscales using twin analysis [34], though these estimates were partially based on twin pairs specifically selected for being at risk of language impairments and may, therefore, not represent the general population. In RAINE, social communication abilities were assessed with a 10-item RAINE-specific broader autism questionnaire [35] at 11 years of age based on parent-report. In order to enhance the similarity of the assessed traits, a short pragmatic composite score (SPC) was constructed based on an item-by-item alignment in both cohorts wherever possible (Table 1, Additional file 1: Figure S1), and consisted of six aligned items. For this, CCC items in ALSPAC were scored as ‘certainly true’ (0), ‘somewhat true’(1), ‘not true’(2), and RAINE broader autism questionnaire items as ‘major problem’(0), ‘minor problem’ (1) or ‘no problem’ (2) resulting in a continuous measure reflecting social communication abilities with a possible range of 0 to 12. Pertinent to this study, this highly left-skewed measure was reverse-coded, thus reflecting social communication problems, in order to facilitate a quantitative analysis of the SPC using a Poisson family model and right-skewed data.

**Table 1:** Item composition of the short pragmatic composite score

**#**	**RAINE**^**a**^	**ALSPAC**^**b**^
1	Tends to talk about subjects that are off topic	Conversation with child tends to go off in unexpected directions
2	Does not have the idea of the need to take turns in conversation	Child talks too much
3	Changes subject indiscriminately	Child will suddenly change topic of conversation
4	Does not introduce subjects and topics appropriately	Child uses terms like he or it without making it clear what talking about
5	Has difficult expressing ideas coherently in a sentence	Sometimes hard to make sense of what child says as it seems illogical/disconnected
6	Has difficulty understanding whole sentences (that is, instructions, directions, general conversation)	Child takes in just one or two words in a sentence so often misinterprets what was said

^a^ – Selected from a 10-item broader autism questionnaire [35] in RAINE.

^b^ – Selected from 38 items representing the pragmatic composite summary scale of the Children’s Communication Checklist [33].

The new measure was generated for analysis purposes only with the aim of capturing most of the shared variation in ALSPAC and RAINE, and has no further diagnostic implication. Furthermore, SPC-based statistical estimates obtained in both samples were only combined using meta-analytic approaches and heterogeneity between statistical estimates was closely monitored using heterogeneity statistics (see below).

The SPC (before reverse-coding) was highly positively correlated with the original pragmatic composite scale (Spearman rank-correlation: ρ = 0.78, *P* <0.0001) and had sufficient internal consistency when investigated in ALSPAC (standardised Cronbach’s α = 0.68) and in RAINE (standardised Cronbach’s α = 0.83).

### Individuals with ASD in ALSPAC and RAINE

Within ALSPAC there is a very small proportion of children with ASD, who were either identified from community paediatric records (National Health Service) or from Education Service databases for the region [36]. Specifically, there were 86 children with ASD at the age of 11 years (prevalence: 62 per 10,000 children). A total of 34 of these children were included within the current study as they were unrelated, of White European descent, and had both CCC/SPC data and genome-wide data. Within RAINE, there are 16 children with clinician diagnosed ASD [37]. Four of these individuals had both genotype and phenotype data available and were included in the current study.

### Genotyping and imputation

ALSPAC children were genotyped using the Illumina HumanHap550 quad chip genotyping platform by 23andMe subcontracting for the Wellcome Trust Sanger Institute, Cambridge, UK and the Laboratory Corporation of America, Burlington, NC, USA. RAINE children were genotyped on an Illumina 660 Quad Array at the Centre for Applied Genomics, Toronto, ON, Canada.

Standard quality control methods were performed in each sample separately and have been previously described [38,39]. In brief, SNPs with a minor allele frequency (MAF) <1%, a call rate <95% or evidence for violations of Hardy-Weinberg equilibrium were removed. Individual samples were excluded on the basis of sex mismatches, minimal or excessive heterozygosity, disproportionate levels of individual missingness, cryptic relatedness, insufficient sample replication and non-European ancestry. In both cohorts, subtle differences in population structure were adjusted for using principal components (Eigenstrat [40]) and genotypic data were imputed using MACH software [41] and phased haplotype data from HapMap CEU (Utah residents with Northern and Western European ancestry from the Centre d'Etude du Polymorphisme Humain collection) individuals (Rel22). Detailed information on genotyping and imputation is given for each cohort in the Additional file 1: Table S1. All reported linkage disequilibrium (LD) measures within this study are based on HapMap CEU (Rel22).

### Genetic association analysis

For the discovery stage of the genome-wide analysis, we investigated 5,584 ALSPAC children with phenotypic information and approximately 2.5 million imputed and genotyped SNPs. The association analysis was performed using a Quasi-Poisson regression approach (‘stats’ R library), which can accommodate for both over- and under-dispersion [42] during the modelling process. Specifically, the SPC was regressed on age, sex - the two most significant ancestry-informative principal components (guided by the evaluation of the respective Eigenvalues using a scree-plot) - and allele dosage. Using a base-line model (without fitting allele dosage) there was a dispersion parameter of ϕ = 1.77 in ALSPAC, which was statistically significant (*P* <0.0001). Regression estimates (β) for allele-dosages represent changes in logcounts of SPC score per effect allele and are reported with their standard errors (SE). SNPs with MAF <1% and poor imputation quality (R^2^ <0.3) were excluded. Subsequently, a genomic-control method was applied to account for potential confounding by population stratification. Devlin and Roeder [43] have developed a method called ‘Genomic control’ that compensates for population stratification by correcting GWAS test statistics, which are presumed to be inflated by a factor λ (where λ can be estimated from a set of unlinked markers). After genomic control (GC)-correction (METAL [44]), we selected the strongest signals from independent loci for *in silico* replication in RAINE. Specifically, we selected a threshold of *P* <1E-05 in order to capture all signals with at least suggestive evidence for genome-wide association. These independent signals and associated LD-regions were identified using the PLINK software ([45], clump options: r^2^ = 0.3, ± 500 kb). In order to assess the overall evidence for association based on all available samples, GC-corrected lead signals from the discovery stage were finally combined with the replication signals using fixed effect inverse-variance meta-analysis (‘rmeta’ R library), while testing for overall heterogeneity using Cochran’s Q-test [46]. Within a fixed effect inverse-variance meta-analysis, evidence for association is combined across studies by computing the pooled inverse variance-weighted beta-coefficient, standard error and z-score [47]. However, in the presence of between-study heterogeneity, the evidence for association might be inflated by fixed effect meta-analysis and it is, therefore, important to test for heterogeneity between samples [47].

In addition to Quasi-Poisson regression, all lead signals were also investigated using Negative Binomial regression (‘MASS’ R library) to examine the robustness of our findings. Negative Binomial regression is an alternative regression technique accounting for the over-dispersion of count data.

In order to prioritise observed SNP signals with evidence for replication in high LD regions, a gene-based association test (Versatile Gene-based Association Study (VEGAS) software [48]) was performed. Gene-based association was empirically assessed based on the *P*-value of all SNPs within a gene, while accounting for LD and the number of SNPs per gene [48].

### Estimation of the proportion of additive phenotypic variance explained by all SNPs together

An estimation of the proportion of additive phenotypic variation explained by all SNPs together was performed using ‘Genome-wide Complex Trait Analysis’ (GCTA) [49]. This method captures the trait variance, which is tagged when all SNPs are considered simultaneously [49]. In this study, GCTA was performed using the full pragmatic composite scale of the CCC (adjusted by age, sex and the first principal components), which is highly correlated with the SPC measure (see above), as well as 464,311 directly genotyped SNPs. In addition, the additive genetic variation was partitioned into individual chromosomes. A quantitative GCTA of the SPC measure itself was not feasible as the measure is highly skewed and transformation was hampered by the limited number of items. Note that small changes in the reported sample numbers compared with the SPC are due to the exclusion of individuals with a relatedness of ≥2.5%. The reason for applying a conservative threshold for the exclusion of family relatives is to avoid the possibility that phenotypic resemblance is due to shared environmental effects or causal effects, which are not tagged by SNPs but captured by pedigree information [49].

### Functional annotation

SNP variation with evidence for replication was investigated *in silico* for the presence of coding variation [50], as well as non-coding variation with high functionality [51] as provided by the ENCODE database.

## Results

### Genome-wide analysis

**Figure 1:**
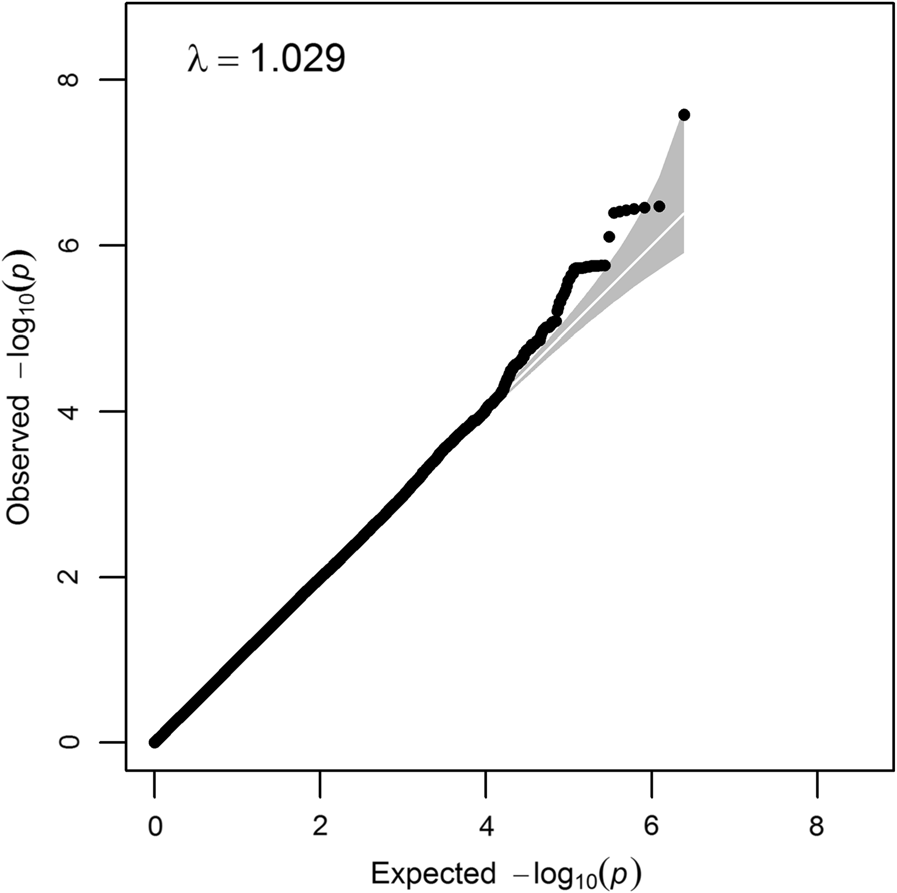
**Quantile-quantile plot for the genome-wide analysis of social communication difficulties in ALSPAC.** The plot is based on genomic-control corrected *P*-values. Black circles depict the observed association signals, the white diagonal line represents the distribution of signals under the null hypothesis and the shaded area corresponds to the 95% confidence interval. A deviation of the observed from the expected distribution of signals is visible. λ, Genomic-control factor.

Characteristics of the discovery and replication samples are presented in Table 2. GWAS in the ALSPAC cohort revealed an excess of association signals beyond chance while detecting little evidence for population stratification (λ_GC_ ≤1.029; Figure 1). The strongest signal was observed at rs4218 within the myosin 1e gene (*MYO1E*) at 15q22.2 (*P* = 2.6E-08, Table 3, Additional file 1: Figure S2) with an increase of 0.11 logcounts of social communication problems per effect allele.

**Table 2:** Study characteristics

				**SPC score**		**Age in years**	
**Study**	**Stage**	**N**^**a**^	**%Male**	**Mean(SD)**	**Range**	**Mean(SD)**	**Range**
ALSPAC	Discovery	5,584	50.3	1.99 (1.88)	0; 12	9.64 (0.12)	9.50; 11.00
RAINE	Replication	1,364	51.8	0.90 (1.69)	0; 11	10.58 (0.20)	9.42; 12.37

^a^ – Samples with genotypic and phenotypic data; SPC, Short pragmatic composite.

**Table 3:** Association results for the lead signals from the discovery analysis

			**Nearest**	**Discovery (N = 5,584)**			**Replication (N = 1,364)**			**Combined (N = 6,948)**		
**SNP**	**Chr**	**E,A**	**Gene**	**EAF**	**β (SE)**^**a**^	***P***^**a**^	**EAF**	**β (SE)**	***P***	**β (SE)**	***P***	**Het-*P***
rs761490	1p32.3	C,G	*TMEM48*	0.24	0.096 (0.022)	9.7E-06	0.23	−0.051 (0.090)	0.57	0.088 (0.021)	3.0E-05	0.11
**rs9257616**	**6p22.1**	**G,A**	***OR2J2***	**0.56**	**0.086 (0.018)**	**3.1E-06**	**0.54**	**0.19 (0.074)**	**0.009**	**0.093 (0.018)**	**2.5E-07**	**0.16**
rs12115663	9p22.3	C,A	*BNC2*	0.86	0.13 (0.027)	4.1E-06	0.87	−0.12 (0.10)	0.23	0.11 (0.026)	3.6E-05	0.018
rs1834180	10q25.1	A,G	intergenic	0.68	0.10 (0.020)	3.3E-07	0.70	0.033 (0.079)	0.68	0.097 (0.019)	4.6E-07	0.40
**rs2352908**	**14q22.1**	**G,T**	**intergenic**	**0.84**	**0.11 (0.026)**	**8.2E-06**	**0.83**	**0.24 (0.10)**	**0.022**	**0.12 (0.025)**	**1.1E-06**	**0.25**
rs11625667	14q24.3	G,A	*TMEM90A*	0.36	0.085 (0.018)	4.3E-06	0.35	−0.049 (0.076)	0.52	0.077 (0.018)	1.7E-05	0.086
rs4218	15q22.2	G,C	*MYO1E*	0.29	0.11 (0.020)	2.6E-08	0.31	−0.027 (0.080)	0.74	0.10 (0.019)	1.0E-07	0.096

^a^ - Genomic-control corrected.

Results are presented for the most significant signals (Genomic-control corrected *P* ≤1E-05) from independent loci during the discovery stage of the analysis. Regression estimates were obtained using Quasi-Poisson regression. All SNPs were imputed and had an imputation quality of 0.90 <R^2^ ≤0.99 (MACH); Replicated signals are indicated in bold. E, Effect allele; A, Alternative allele; EAF, Effect allele frequency; Het-*P*, Heterogeneity *P-*value.

Selecting the strongest signals from recent ASD GWAS, we furthermore investigated whether the allele conferring risk to ASD also increased the expression of social communication difficulties in the general population, as captured by the SPC score within the ALSPAC sample (Additional file 1: Table S2). This analysis did not identify evidence for novel ASD QTL spanning the entire spectrum, but confirmed the previously identified association between social communication traits in ALSPAC and common ASD risk variants at 5p14 [13]. Specifically, this involved the association with variation at the ASD high-risk locus rs4307059 [12] (β = 0.066 (0.019), *P* = 0.00041). In addition, we observed evidence for association at rs10038113 (β = −0.0391 (0.018), *P* = 0.032), a second ASD risk locus [26], which resides approximately 65 kb upstream of rs4307059 at 5p14. The association at rs4307059 was attenuated (β = 0.067 (0.025), *P* = 0.0063) and the association at rs10038113 abolished (β = −0.0042 (0.023), *P* = 0.86) when variant analyses were conditioned on each other, suggesting that these signals are not independent. Together the association findings at 5p14 thus strengthen the validity of the utilised SPC score, that is, the extent to which the SPC score captures ASD-related social communication symptoms.

**Figure 2:**
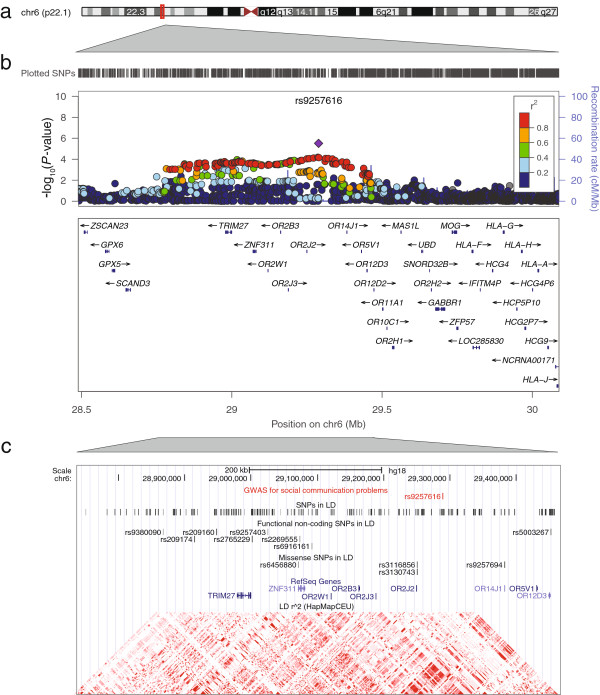
**Association plot for the association between social communication problems and common variation at 6p22.1. a).** Chromosome ideogram for chromosome 6. **b)**. Regional association plot for rs9257616 on chromosome 6p22.1. Directly genotyped and imputed variants are depicted by filled circles according to their GWAS *P*-value (−log10 *P*-value) and genomic position (NCBI Build 36). The local LD structure is reflected by HapMap CEU (Rel 22) recombination rates (blue line). The LD (r^2^) between the lead variant and surrounding SNPs is indicated by the colour code. **c)**. Detailed genomic region near rs9257616 on chromosome 6p22.1 with variants in LD (r^2^ >0.3) including non-coding functional (Regulome score ≤2) and missense variation. The LD (r^2^) between the lead variant and surrounding SNPs is indicated by the colour code (0 (white)-1(black)). The local LD structure is reflected by HapMap CEU (Rel 22) r^2^ –based haplotype blocks. GWAS, genome-wide association studies; LD, linkage disequilibrium; SNPs, single nucleotide polymorphisms.

**Figure 3:**
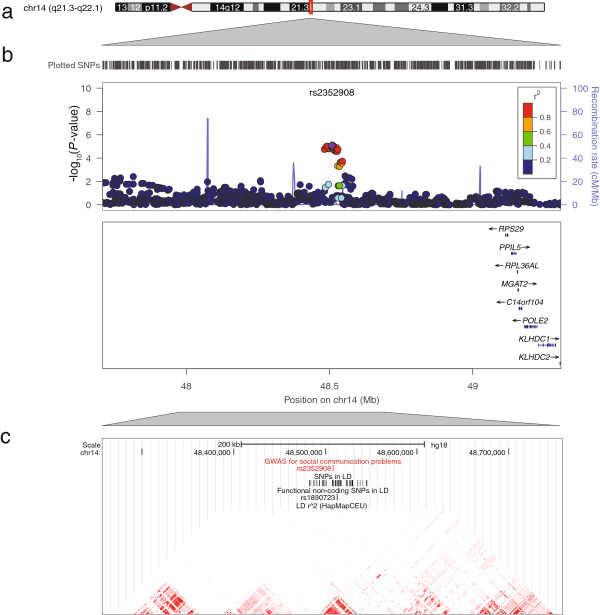
**Association plot for the association between social communication problems and common variation at 14q22.1. a).** Chromosome ideogram for chromosome 14. **b)**. Regional association plot for rs2352908 on chromosome 14q22.1. Directly genotyped and imputed variants are depicted by filled circles according to their GWAS *P*-value (−log10 *P*-value) and genomic position (NCBI Build 36). The local LD structure is reflected by HapMap CEU (Rel 22) recombination rates (blue line). The LD (r^2^) between the lead variant and surrounding SNPs is indicated by the colour code. **c)**. Detailed genomic region near rs2352908 on chromosome 14q22.1 with variants in LD (r^2^ >0.3) including non-coding functional variation (Regulome score ≤2). The LD (r^2^) between the lead variant and surrounding SNPs is indicated by the colour code (0 (white)-1(black)). The local LD structure is reflected by HapMap CEU (Rel 22) r^2^ –based haplotype blocks. GWAS, genome-wide association studies; LD, linkage disequilibrium; SNPs, single nucleotide polymorphisms.

In an attempt to replicate the association at rs4218 as well as six further signals from independent loci (*P* <1E-05), we investigated these variants *in silico* in RAINE. Two of these variants showed association with social communication problems with the same direction of effect as observed in ALSPAC (Table 3), including rs9257616 near the olfactory receptor 2 J2 gene (*OR2J2*) at 6p22.1 and rs2352908 within an intergenic interval at 14q22.1 (Figures 2 and 3 respectively). Association signals at these SNPs reached suggestive evidence for genome-wide association within the combined cohort sample, while expressing little evidence for heterogeneity: rs9257616, β = 0.093(0.018), meta-*P* = 2.5E-07, Het-*P* = 0.16 and rs2352908, β = 0.12(0.025), meta-*P* = 1.1E-06, Het-*P* = 0.25. Alternative statistical modelling using negative binomial regression confirmed the nature of these findings (Additional file 1: Table S3). There was however no support for an association at rs4218 in RAINE, our strongest signal from the discovery analysis (*P* = 0.74, Table 3).

### GCTA

Further support for the contribution of common variation to the genetic architecture of social communication traits was provided through the quantification of the proportion of the phenotypic variance in pragmatic composite scores in ALSPAC, which is accounted for by all genotyped SNPs together (narrow sense heritability h^2^ (SE) = 0.18 (0.066), *P* = 0.003, N = 5,244). The highly correlated CCC-based pragmatic composite score was utilised as a proxy for the SPC score (as the SPC score is a subset of the pragmatic composite score), since the SPC measure itself could not be subjected to GCTA (see Methods).

**Figure 4:**
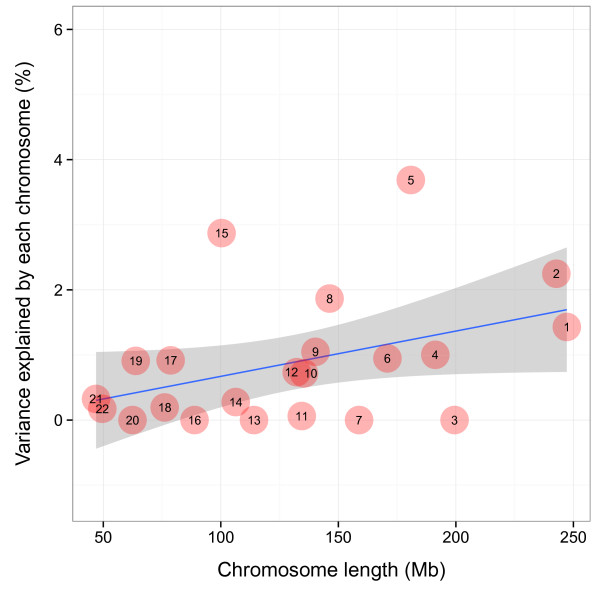
**Estimate of the proportion of genetic variance in social communication difficulties explained by each chromosome.** Numbers reflect individual chromosomes; the blue line indicates the linear regression of chromosome length on the proportion of variation explained (adjusted regression R^2^ = 0.12, *P* = 0.06); confidence intervals are indicated in grey.

We subsequently partitioned pragmatic composite score-related genetic variance into individual chromosomes, fitting all chromosomes simultaneously, and observed a trend for a linear relationship between chromosome length and explained variance supporting a polygenic inheritance model (adjusted regression R^2^ = 0.12, *P* = 0.06). However, some chromosomes, including 5, 8 and 15, may explain more phenotypic variance than predicted by the linear model (Figure 4).

### Annotation of functionality

The LD structure within the vicinity of rs9257616 at 6p22.1 is complex and far reaching (LD-based gene region: approximately 707 kb; Figure 2). Specifically, the genomic region contains a cluster of genes (*TRIM27, ZNF311, OR2W1, OR2B3, OR2J3, OR2J2, LOC651503* (inferred pseudogene *OR2U1P*)*, OR214J1, OR5V1, OR12D3*) among which *TRIM27* provided the strongest evidence for gene-based association locally (Additional file 1: Table S4). The candidacy of *TRIM27* was strengthened by the presence of functional non-coding variation (Figure 2) within the vicinity of the gene (rs2765229: r^2^ = 0.91, rs9380090: r^2^ = 0.41, rs9257403: r^2^ = 0.43). According to the ENCODE database annotation (Additional file 1: Table S5), this variation is likely to affect the binding of various proteins, is related to histone modifications to DNA and linked to the expression of *TRIM27* in monocytes*.* However, variation at rs9257616 was also in LD (r^2^ >0.3) with missense mutations in *OR2J2* (rs3116856, V(GTT) → A(GCT), r^2^ = 0.74; rs3130743,T(ACC) → A(GCC), r^2^ = 0.91), the zinc finger protein *ZNF311* (rs6456880, K(AAG) → Q(CAG), r^2^ = 0.58) and *OR14J1* (rs9257694, M(ATG) → T(ACG), r^2^ = 0.85).

The intergenic region at 14q22.1 (LD-based gene region: 62 kb; Figure 3), which harbours rs2352908, did not contain any genes within the vicinity of the signal, nor within a wider genomic region (+/− 500 kb). The closest locus, the ribosomal protein S29 gene (*RPS29*) residing 606 kb downstream of the SNP, is separated from the variant through a recombination peak. However, there was ENCODE-based evidence for variation within a nearby functional non-coding site (rs1890723), which was in complete LD with rs2352908 (r^2^ = 1) and linked to HNF4-based transcription regulation (Additional file 1: Table S5).

### Phenotypic characterisation of signals

Analyses taking into account potential ASD-related covariates (Additional file 1: Tables S6 and S7) revealed that variation at rs2352908 was associated with an increased probability of hearing problems, in both ALSPAC (odds ratio (OR) with SE = 1.48 (0.24), *P* = 0.016) and RAINE (OR = 1.49 (0.29), *P* = 0.038), which was strongest when analyses were combined (OR = 1.49 (0.18), *P* = 0.0014). In addition, we observed weaker evidence for association between rs9257616 and internalising problems within the combined cohorts (OR = 1.17 (0.081), *P* = 0.022). Both signals were marginally attenuated when analyses were adjusted for hearing problems and internalising problems, respectively (Additional file 1: Table S8). We found no evidence for the influence of other potential covariates, including verbal and performance intelligence quotient (IQ) scores, mother’s educational level and conduct problems. Only the combined association signal between variation at rs2352908 and hearing problems would remain significant after adjustment for multiple testing.

## Discussion

This genome-wide study represents a large quantitative analysis of social communication problems in the general population, analysing a total of 6,948 children of White European descent, and provided support for the implication of common variation in the genetic architecture of these traits. Two of our seven top single SNP signals at 6p22.1 (rs9257616, meta-*P* = 2.5E-07) and at 14q22.1 (rs2352908, meta-*P* = 1.1E-06) were replicated within an independent sample of 11-year-old children with comparable measures from Western Australia, although they fell short of reaching conventional levels of genome-wide association. Overall, approximately a fifth (approximately 18%) of the variation in social communication difficulties was explained by joint additive genetic effects of common SNPs (MAF >1%), and our findings support a polygenic mode of inheritance.

Intriguingly, the observed GCTA heritability estimates for social communication traits in the general population are highly similar to recently reported GCTA heritability estimates in relatives of ASD probands [52], strengthening the molecular support for an underlying broader autism phenotype. Based on analyses of the Simons Simplex Collection and the Autism Genome Project samples (contrasting two population control samples), substantial additive genetic influences were identified in fathers (h^2^ = 0.20 to 0.52), mothers (h^2^ = 0.20 to 0.37) and unaffected siblings (h^2^ = 0.16) [52]. The heritability estimates in our study are, however, smaller than previous twin study reports on autistic traits (h^2^ = 0.36 to 0.87 [3-9]) as GCTA estimates reflect only the lower limit of the narrow-sense heritability and depend on the assumption that causal variation is sufficiently represented through the selected set of genotyped SNPs [49]. As such, GCTA estimates may account on average only for about half of the heritability observed within twin designs [53].

The strongest replicated single SNP signal has been identified within the olfactory receptor gene cluster at 6p22.1, which is part of the broader major histocompatibility complex (MHC) region. On a larger scale, this genomic area has been previously related to autistic symptoms through association and linkage of the HLA-A2 class I allele with ASD [54] (approximately 768 kb downstream of the signal). The extensive LD across the MHC region, however, hampers the evaluation of a single locus candidacy. Both regional gene-based analysis in ALSPAC and the presence of functional non-coding variation pointed to *TRIM27* (OMIM: 602165 [55]) as a candidate locus, which encodes a member of the tripartite motif (TRIM) family. TRIM27 is a DNA-binding protein associated with the nuclear matrix and interacts with methyl-CpG-binding domain (MBD) proteins [56], including MBD2, MBD3 and MBD4, and rare autism-specific protein-changing alterations have been observed both in *MBD3* and *MBD4*[57]. Social communication related variation at 6p22.1 may, however, also involve one of the many OR loci or the uncharacterised *ZNF311* gene, as protein altering variation at these sites has been found in LD with rs9257616. Furthermore, the replicated signals at 14q22.1 might be of interest as this association was supported by secondary analyses, including hearing impairments in both ALSPAC and RAINE. It might be speculated that this may reflect the non-pathological equivalent of an increased frequency of auditory symptoms, such as auditory filtering [58,59] or impairment in hearing [60], which is often observed in individuals with ASD.

Partitioning of the genetic variance into chromosomes supported, furthermore, a polygenic model of inheritance, which may involve multiple loci of weak effect. This is consistent with the proposed role of common variation in ASD [24], which is likely to affect risk to disease through a (log)-additive combination of multiple loci of small effect, but also the implication of common variation within behavioural traits, such as cognitive ability [61]. It is also possible that these findings may extrapolate to other ages, with evidence from both ALSPAC [11,62] and RAINE [63] suggesting that pragmatic language skills are stable across development. However, much larger sample sizes might be required to detect loci of modest individual effects, and failure to replicate or reach conventional levels of genome-wide association may not necessarily preclude the existence of genuine (but weak) loci. In light of this, also the strongest association signals within ALSPAC, including variation at 15q22.2, although not replicated in the smaller RAINE sample, might be re-visited in future studies. In general, chromosome 15 harbours a large amount of common social communication related genetic variation, which is larger than expected by its size. More specifically, the signal at 15q22.2 was also in LD with variants at *RNF111,* a gene which has been recently implicated in Asperger disorder through association [25]. However, even if this common signal is genuinely implicated in the genetic architecture of social communication traits, the underlying genetic mechanisms are likely to be different at each end of the autistic continuum, as we found no evidence that the Asperger-related single SNP variation contributes to the association signal within ALSPAC (data not shown). In addition, our findings strengthened the evidence for the presence of an ASD QTL at 5p14. Besides the signal reported by Wang and colleagues [12], which has been previously related to the expression of social communication traits in ALSPAC [13], we also observed association with a second 5p14 signal, identified by Ma and colleagues [26]. Conditional analysis suggested that both SNPs refer to the same underlying causal variation, thus linking both loci to the recently proposed disease mechanism involving the transcription of non-coding RNA [64].

Common genetic effects are implicated within many quantitative traits through a polygenic mode of inheritance [61,65]. While genome-wide genetic association screens for anthropometric phenotypes, such as height, have been, however, highly successful [65], genetic association studies involving complex behavioural traits have so far failed to robustly identify single SNP association signals [61,66]. Our discovery sample (Genetic power calculator; http://pngu.mgh.harvard.edu/~purcell/gpc/) had sufficient power (>0.83) to detect genetic effects explaining as little as 0.7% of the phenotypic variance, assuming for simplicity a normally distributed phenotype and complete LD between marker and disease locus, in addition to a type I error of α = 5E-08. However, the true inherent power of our study might have been compromised as parent reports of social communication difficulties in children represent a far noisier and less reliable quantitative data source than comparable anthropometric phenotypes [65], making additional data cleaning and analysis steps indispensable. Within our study, we therefore selected a highly similar phenotype definition in both the discovery and the replication cohort. Problems in social communication skills as assessed by the newly defined measure are closely related to difficulties in conversational skills, such as turn taking, topic maintenance and discourse coherence. The newly defined measure had sufficient internal consistency, was highly correlated with the original CCC pragmatic composite scale [33] and consistent with a previously reported association between social communication traits and common variation at an ASD risk locus at 5p14 [13]. Furthermore, for pragmatic abilities, parent-report has been shown to be a more accurate measurement than self-report, primarily because this method allows for the assessment of communication in a variety of contexts [67]. In addition, we selected a Quasi-Poisson regression approach, which specifically modelled the skewed phenotypic data distribution without information loss through transformation. As such, these “power-boosting” measures may have increased the true underlying power of our study through a reduction in measurement noise. Indeed, within the specific context of GWAS of quantitative cognitive/behavioural traits our findings stand out as we identified evidence for social communication-related genetic variation through replication. However, within the general context of GWAS studies, the reported single SNPs signals reached only suggestive levels of genome-wide association and, even under the “power-boosting” circumstances, many more samples might be required to identify common genetic association signals with high confidence. Furthermore, the limited number of items that comprised the SPC (n = 6), may have captured only selected aspects of social communication problems. Thus, further replication efforts may require similar item alignments in order to enhance the comparability of findings across studies.

## Conclusion

Our study provided evidence that common genetic variation jointly accounts for approximately a fifth of the phenotypic variation in social communication difficulties in the general population. There was furthermore support for single SNP association at 6p22.1 and 14q22.1 based on replication in independent samples, although these signals fell short of reaching conventional levels of genome-wide significance. Together our findings suggest that common genetic variation contributes to the genetic architecture of social communication traits and may indeed involve some individual loci with genetic effects large enough to be detectable in association screens.

### Availability of supporting data

Supplementary information is provided as Additional material.

## Abbreviations

ASD: Autism spectrum disorders; ALSPAC: Avon Longitudinal Study of Parents and their Children; CCC: Children’s Communication Checklist; CNV: Copy number variation; DNA: Deoxyribonucleic acid; GC: Genomic control; GCTA: Genome-wide complex trait analysis; GWAS: Genome-wide association study; KEMH: King Edward Memorial Hospital; LD: Linkage disequilibrium; MAF: Minor allele frequency; MBD: Methyl-CpG-binding domain; MHC: Major histocompatibility complex; OR: Odds ratio; QTL: Quantitative trait locus; SE: Standard error; SNP: Single nucleotide polymorphism; SPC score: Short Pragmatic Composite Score.

## Competing interests

The authors declare that they have no competing interests.

## Authors’ contributions

BSP, AJOW, WQA and NMW carried out the statistical analysis. BSP, DME, JPK, SMR, WLM and NMW were involved in the preparation of the genotype information. BSP, AJOW, CEP and GDS participated in the design of the study. BSP, AJOW, WQA, JTG, KW, NJT, DMW, JPK, JG, HH, CEP and GDS helped to draft the manuscript. All authors read and approved the final manuscript.

## Supplementary Material

Additional file 1: Table S1 Cohort-specific genotyping and imputation information. **Table S2.** Investigation of GWAS ASD association signals within the general population (ALSPAC) using the SPC. **Table S3.** Association results for the lead signals from the discovery analysis (Negative binomial regression). **Table S4.** Gene-based analysis of loci at 6p22.1. **Table S5.** Functional characterisation of non-coding variation in linkage disequilibrium with rs9257616 and rs2352908. **Table S6.** Association between replicated signals and potential covariates. **Table S7.** Association between replicated signals and intelligence. **Table S8.** Association for replicated lead signals with and without adjustment for potential covariates. **Figure S1.** Histogram of the short pragmatic composite score (SPC) in ALSPAC before reverse-coding. **Figure S2.** Regional association plot (Build 36) for the top 5 independent regions in the ALSPAC discovery cohort, which did not achieve replication, ordered by significance in the discovery analysis. Click here for file
