# Supplementary material for: Common variation contributes to the genetic architecture of social communication traits
Source: Mol Autism. 2013 Sep 18;4:34. doi: 10.1186/2040-2392-4-34 (PMC3853437; doi:10.1186/2040-2392-4-34)
Supplement: Additional file 1: Table S1 — Cohort-specific genotyping and imputation information. Table S2. Investigation of GWAS ASD association signals within the general population (ALSPAC) using the SPC. Table S3. Association results for the lead signals from the discovery analysis (Negative binomial regression). Table S4. Gene-based analysis of loci at 6p22.1. Table S5. Functional characterisation of non-coding variation in linkage disequilibrium with rs9257616 and rs2352908. Table S6. Association between replicated signals and potential covariates. Table S7. Association between replicated signals and intelligence. Table S8. Association for replicated lead signals with and without adjustment for potential covariates. Figure S1. Histogram of the short pragmatic composite score (SPC) in ALSPAC before reverse-coding. Figure S2. Regional association plot (Build 36) for the top 5 independent regions in the ALSPAC discovery cohort, which did not achieve replication, ordered by significance in the discovery analysis. [file 2040-2392-4-34-S1.docx]

**Common variation contributes to the genetic architecture of social communication traits**

Beate St Pourcain PhD, Andrew J.O. Whitehouse PhD, Wei Q. Ang MSc, Nicole M. Warrington BSc, Joseph T. Glessner MS, Kai Wang PhD, Nicholas J. Timpson PhD, David M. Evans PhD, John P. Kemp MSc, Susan M. Ring PhD, Wendy L. McArdle PhD, Jean Golding DSc, Hakon Hakonarson PhD, Craig E. Pennell PhD, George Davey Smith DSc

**Additional Material**

1. **Additional Tables**

Table S1: Cohort-specific genotyping and imputation information

Table S2: Investigation of GWAS ASD association signals within the general population (ALSPAC) using the SPC

Table S3: Association results for the lead signals from the discovery analysis (Negative binomial regression)

Table S4: Gene-based analysis of loci at 6p22.1

Table S5: Functional characterisation of non-coding variation in linkage disequilibrium with rs9257616 and rs2352908

Table S6: Association between replicated signals and potential covariates

Table S7: Association between replicated signals and intelligence

Table S8: Association for replicated lead signals with and without adjustment for potential covariates

1. **Additional Figures**

Figure S1: Histogram of the short pragmatic composite score (SPC) in ALSPAC before reverse-coding.

Figure S2: Regional association plot (Build 36) for the top 5 independent regions in the ALSPAC discovery cohort, which did not achieve replication, ordered by significance in the discovery analysis

**Additional Tables**

**Table S1**: Cohort-specific genotyping and imputation information

| **Sample** | **Origin** | **N^a^** | **Genotyping**  **platform** | **Genotyping quality control** | | | | **N SNPs before** | **Imputation software** | **NCBI**  **Build** |
| --- | --- | --- | --- | --- | --- | --- | --- | --- | --- | --- |
|  |  |  |  | **HWE-p** | **SNP call rate** | **Sample Call rate** | **MAF** | **imputation** |  |  |
| ALSPAC | British | 8365 | Illumina HumanHap550 | 5.0E-07 | 0.95 | 0.97 | 0.01 | 464,311 | MACH | 36 |
| RAINE | Australian | 1494 | Illumina 660 Quad Array | 5.7E-07 | 0.95 | 0.97 | 0.01 | 535,632 | MACH | 36 |

a – Independent individuals of European descent with genome-wide genotype data after quality control (irrespective of available phenotypic information)

MAF – Minor allele frequency; HWE-p –Hardy Weinberg *p*-value

**Table S2**: Investigation of ASD GWAS signals within the general population (ALSPAC) using the SPC

|  |  |  | **Nearest** | **ASD association signals** | | | **ALSPAC GWAS (SPC)** | | |
| --- | --- | --- | --- | --- | --- | --- | --- | --- | --- |
| **SNP** | **Chr** | **E^a^,A** | **gene** | **EAF^b^** | **Effect** | **Meta-p** | **EAF** | **β(SE)^c^** | **p** |
| rs10038113 | 5p14.1 | T,C | intergenic | 0.59 | protective^d^ | 3.4E-06 | 0.60 | -0.0391(0.018) | **0.032** |
| rs4307059 | 5p14.1 | T,C | intergenic | 0.62-0.65 | risk^e^ | 2.1E-10 | 0.62 | 0.066(0.019) | **0.00041** |
| rs10513025 | 5p15.2 | C,T | *TAS2R1,SEMA5A* | - | protective^f^ | 2.1E-07 | 0.040 | 0.00050(0.053) | 0.99 |
| rs4703129 | 5q21.1 | A,C | *intergenic* | 0.38-0.41 | not reported^g^ | 9.7E-07 | 0.41 | 0.014(0.018) | 0.44 |
| rs4141463 | 20p12.1 | A,G | *MACROD2* | 0.43 | protective^h^ | 3.7E-08 | 0.40 | -0.026(0.019) | 0.18 |

a – As reported in the ASD GWAS

b – Within diseased population

c – Genomic-control corrected

d – Ma et al., 2009[1]

e – Wang et al., 2009[2]

f – Weiss et al., 2009[3]

g – Salyakina et al., 2010[4]; no effect allele was reported

h – Anney et al, 2010[5]

The selected SNPs represent the strongest association signals from recent ASD GWAS. Population-based results are presented for the Short Pragmatic Composite score (SPC) using a Quasi-Poisson regression approach. E– Effect allele, A – Alternative allele, EAF – Effect allele frequency, Meta p – *P*-value from meta-analysis as reported in the ASD GWAS; ASD – Autism spectrum disorder

**Table S3:** Association results for the lead signals from the discovery analysis (Negative binomial regression)

|  |  |  | **Nearest** | **Discovery (N=5584)** | | | **Replication (N=1364)** | | | **Combined (N=6948)** | | |
| --- | --- | --- | --- | --- | --- | --- | --- | --- | --- | --- | --- | --- |
| **SNP** | **Chr** | **E,A** | **gene** | **EAF** | **β (SE)^a^** | **p^a^** | **EAF** | **β (SE)** | **p** | **β (SE)** | **p** | **Het-p** |
| rs761490 | 1p32.3 | C,G | *TMEM48* | 0.24 | 0.097(0.022) | 1.3E-05 | 0.23 | -0.054(0.097) | 0.58 | 0.089(0.022) | 3.5E-05 | 0.13 |
| **rs9257616** | **6p22.1** | **G,A** | ***OR2J2*** | **0.56** | **0.087(0.019)** | **2.6E-06** | **0.54** | **0.20(0.079)** | **0.010** | **0.093(0.018)** | **2.5E-07** | **0.15** |
| rs12115663 | 9p22.3 | C,A | *BNC2* | 0.86 | 0.13(0.027) | 3.2E-06 | 0.87 | -0.11(0.11) | 0.33 | 0.11(0.026) | 1.7E-05 | 0.042 |
| rs1834180 | 10q25.1 | A,G | intergenic | 0.68 | 0.10(0.02) | 2.8E-07 | 0.70 | 0.03(0.086) | 0.73 | 0.098(0.019) | 3.6E-07 | 0.41 |
| **rs2352908** | **14q22.1** | **G,T** | **intergenic** | **0.84** | **0.11(0.025)** | **7.7E-06** | **0.83** | **0.22(0.11)** | **0.036** | **0.12(0.025)** | **1.3E-06** | **0.32** |
| rs11625667 | 14q24.3 | G,A | *TMEM90A* | 0.36 | 0.084(0.019) | 6.3E-06 | 0.35 | -0.040(0.081) | 0.62 | 0.078(0.018) | 1.8E-05 | 0.14 |
| rs4218 | 15q22.2 | G,C | *MYO1E* | 0.29 | 0.11(0.02) | 3.9E-08 | 0.31 | -0.025(0.086) | 0.77 | 0.10(0.02) | 1.3E-07 | 0.12 |

a - Genomic-control corrected

Results are presented for the most significant signals (Genomic-control corrected P ≤ 1E-05) from independent loci during the discovery stage of the analysis, which were re-analysed using Negative Binomial regression. Regression estimates (β) represent changes in log counts of SPC score per increase in effect allele. All SNPs had an imputation quality of 0.90 < R^2^ < 0.99 (MACH); Replicated signals are indicated in bold. E – Effect allele, A – Alternative allele, EAF – Effect allele frequency, Het-p – Heterogeneity *p*-value

**Table S4:** Gene-based analysis of loci on chromosome 6p22.1

| **Gene** | **Position(hg18)** | **N SNPs** | **Gene-based p** | **Best-SNP** | **SNP-based p** |
| --- | --- | --- | --- | --- | --- |
| *TRIM27* | chr6:28978757-28999747 | 114 | 0.00025 | rs4713186 | 0.00011 |
| *OR2J3* | chr6:29187646-29188582 | 90 | 0.00037 | rs3130778 | 0.00013 |
| *LOC651503* | chr6:29338458-29339835 | 76 | 0.00050 | rs9257616 | 3.08E-06 |
| *OR2J2* | chr6:29249289-29250330 | 81 | 0.00054 | rs9257616 | 3.08E-06 |
| *OR2B3P* | chr6:29162062-29163004 | 94 | 0.00092 | rs3130778 | 0.00013 |
| *OR2W1* | chr6:29119968-29120931 | 97 | 0.00097 | rs6456880 | 0.00022 |
| *ZNF311* | chr6:29070572-29081016 | 104 | 0.00105 | rs6901599 | 0.00014 |
| *OR5V1* | chr6:29430985-29432033 | 217 | 0.030 | rs9257693 | 0.00020 |
| *OR12D3* | chr6:29449178-29451047 | 216 | 0.037 | rs12197616 | 0.00074 |

Gene-based p-values are based on 1000000 simulations as implemented in VEGAS [6]; LD – Linkage disequilibrium; The *OR214J1* was not contained within the list of reference genes analysed by VEGAS. All reported best SNPs are in LD with rs9257616 (r^2^>0.5). Selected loci are based on a LD based gene region of ~707 kb near rs9257616

**Table S5:** Functional characterisation of non-coding variation in linkage disequilibrium with rs9257616 and rs2352908

| **SNP** | **Chr** | **r^2^** | **Gene** | **Reg** | **eQTL** | **TF motif** | **Histone modification (ChiP-seq)** | **Protein binding (ChiP Seq)** | **DNase Seq** |
| --- | --- | --- | --- | --- | --- | --- | --- | --- | --- |
| rs9380090 | 6p22.1 | 0.41 | *TRIM27* | 1f | TRIM27 (Monocytes) | - | Yes(Multiple) | - | Yes(Helas3) |
| rs2765229 | 6p22.1 | 0.91 | *TRIM27* | 1f | TRIM27 (Monocytes) | Nkx2-6, Nkx2-4 | Yes(Multiple) | - | Yes(K562) |
| rs9257403 | 6p22.1 | 0.43 | *TRIM27* | 1f | TRIM27 (Monocytes) | - | Yes(Multiple) | Yes(Multiple) | Yes(Multiple) |
| rs209174 | 6p22.1 | 0.91 | *LOC401242* | 2b | - | IRF3 | Yes(Multiple) | Yes(Multiple) | Yes(Multiple) |
| rs209160 | 6p22.1 | 0.93 | *LOC401242* | 2b | - | TCF11 | Yes(Multiple) | Yes (HepG2) | Yes(UrotsaUt189) |
| rs2269555 | 6p22.1 | 0.56 | *ZNF311* | 2b | - | Multiple motifs | Yes(Multiple) | Yes(Multiple) | Yes(Multiple) |
| rs6916161 | 6p22.1 | 0.60 | *ZNF311* | 2b | - | HMGIY | Yes(Multiple) | Yes(Multiple) | Yes(Multiple) |
| rs5003267 | 6p22.1 | 0.75 | *OR12D3* | 2b | - | Multiple motifs | Yes(Multiple) | Yes(Multiple) | Yes(Multiple) |
| rs1890723 | 14q22.1 | 1 | *-* | 2c | - | HNF4, HNF4A | Yes(Multiple)) | HNF4A(Caco2) | Yes(Multiple) |

r^2^ – Linkage disequilibrium with rs9257616 and rs2352908 respectively; *Annotation is only given for variants with strong evidence for functional non-coding variation* (ENCODE database annotation [7]: Regulome codes 1 and 2; 1 - Likely to affect binding of a protein to DNA and linked to expression of a gene target, 2 - Likely to affect binding of a protein to DNA); Reg – Regulome database score: 1f - eQTL + TF binding / DNase peak; 2b - TF binding + any motif + DNase footprint + DNase peak; 2c - TF binding + matched TF motif + DNase peak; eQTL - Expression quantitative trait locus related to SNP variation; TF – Transcription factor binding motif; ChIP-seq - Chromatin immunoprecipitation (ChIP) with massively parallel DNA sequencing to identify the binding sites of DNA-associated proteins and histone modifications; Dnase Seq - DNase I hypersensitive sites sequencing; Information on cell lines are given in parentheses (HeLa-S3 – Cervical cancer cell line; K562 – Leukemia cell line; HepG2 – Liver carcinoma cell line; Caco-2: Colorectal adenocarcinoma cell line); Multiple – Multiple cell lines

**Table S6:** Association between replicated signals and potential covariates

|  |  | **Discovery^c^** | | | **Replication^d^** | | | | **Combined** | | | |
| --- | --- | --- | --- | --- | --- | --- | --- | --- | --- | --- | --- | --- |
| **Covariate** | **SNP^b^** | **N** | **OR(SE)** | **p** | | **N** | **OR(SE)** | **p** | **N** | **OR(SE)** | **p** | **Het-p** |
| Maternal education (R:high) | rs9257616_G | 7407 | 1.02(0.039) | 0.56 | | 1494 | 0.90(0.073) | 0.20 | 8901 | 1.00(0.035) | 0.98 | 0.16 |
|  | rs2352908_G | 7407 | 1.13(0.06) | 0.020 | | 1494 | 0.95(0.098) | 0.64 | 8901 | 1.09(0.051) | 0.064 | 0.14 |
| Conduct problems (R: low)^a^ | rs9257616_G | 5752 | 1.00(0.075) | 0.97 | | 1131 | 1.35(0.19) | 0.031 | 6883 | 1.07(0.071) | 0.32 | 0.057 |
|  | rs2352908_G | 5752 | 1.25(0.14) | 0.040 | | 1131 | 0.861(0.15) | 0.39 | 6883 | 1.13(0.10) | 0.19 | 0.072 |
| Internalising problems (R: low)^a^ | **rs9257616_G** | **5737** | **1.16(0.09)** | **0.064** | | **1131** | **1.24(0.19)** | **0.16** | **6868** | **1.17(0.081)** | **0.022** | **0.69** |
|  | rs2352908_G | 5737 | 1.18(0.13) | 0.13 | | 1131 | 0.98(0.19) | 0.92 | 6868 | 1.13(0.11) | 0.20 | 0.41 |
| Hearing problems (R: low)^a^ | rs9257616_G | 5609 | 0.98(0.1) | 0.88 | | 1364 | 1.09(0.14) | 0.54 | 6973 | 1.02(0.084) | 0.80 | 0.56 |
|  | **rs2352908_G** | **5609** | **1.48(0.24)** | **0.016** | | **1364** | **1.49(0.29)** | **0.038** | **6973** | **1.49(0.18)** | **0.0014** | **0.99** |

a – Adjusted for age and sex in the total sample, adjusted for age only in the female subsample

b – Coded with respect to the risk allele

c – In ALSPAC, information on maternal education was obtained using questionnaires at 32 weeks gestation and ranked as follows: ‘Below O-level’/‘O-level’(low level of maternal education) and ‘Above O-level’(high level of maternal education), O-levels are UK school-leaving qualifications taken at age 16; Mother-reported conduct and internalising problems in children were assessed at 10 years of age using the Strengths-and-Difficulties Questionnaire (SDQ)[8] and dichotomised into high and low scorers according to the recommended banding [8] ; Hearing thresholds in children for conventional frequencies were measured using air and bone conduction (GSI 61 clinical audiometer and TDH50P headphones) and classified into hearing problems (Mild or moderate uni- or bilateral hearing impairment ) versus bilateral normal hearing

d – In RAINE, information on maternal education was obtained using questionnaires at 34 weeks gestation and assessed with the question (‘Completed secondary school’ versus ‘Did not complete secondary school’); Mother-reported conduct and internalising problems in children were assessed at 10 years of age using the SDQ[8] and dichotomised into high and low scorers according to the recommended banding [8]; Hearing problems in children were based on parent report at 8 years of age and assessed with the question (‘Ever been diagnosed with a hearing problem’)

Regression estimates were obtained using Logistic regression. Replicated signals and signals with a trend for replication are indicated in bold. R – Reference level, OR – Odds ratio, Het-p – Heterogeneity *p*-value

**Table S7:** Association between replicated signals and intelligence

|  |  | **Discovery^c^** | | | **Replication^d^** | | | **Combined** | | | |
| --- | --- | --- | --- | --- | --- | --- | --- | --- | --- | --- | --- |
| **Covariate** | **SNP^b^** | **N** | **β(SE)** | **p** | **N** | **β(SE)** | **p** | **N** | **β(SE)** | **p** | **Het-p** |
| Verbal IQ (Z-scores)^a^ | rs9257616_G | 5540 | -0.017(0.019) | 0.37 | 1103 | 0.0004(0.044) | 0.99 | 6643 | -0.014(0.018) | 0.41 | 0.71 |
|  | rs2352908_G | 5540 | -0.041(0.026) | 0.12 | 1103 | -0.068(0.69) | 0.24 | 6643 | -0.041(0.026) | 0.12 | 0.97 |
| Performance IQ (Z-scores)^a^ | rs9257616_G | 5535 | -0.012(0.019) | 0.55 | 1184 | 0.026(0.043) | 0.54 | 6719 | -0.0053(0.018) | 0.76 | 0.42 |
|  | rs2352908_G | 5535 | -0.039(0.026) | 0.14 | 1184 | -0.001(0.056) | 0.99 | 6719 | -0.032(0.024) | 0.18 | 0.54 |

a – Adjusted for sex in total sample, unadjusted for females

b – Coded with respect to the risk allele

c – Verbal and performance intelligence quotient scores in ALSPAC children were measured with the Wechsler-Intelligence-Scale for Children (WISC-III)[9] at 9 years of age

d – Verbal IQ scores in RAINE were based on the Peabody Picture Vocabulary Test – Revised[10] at age 10 years, and Performance IQ scores were based on block design subtest of the WISC-III at age 8 years.

Regression estimates were obtained using Ordinary Least Squared regression. Het-*p* – Heterogeneity p-value

**Table S8:** Association for replicated lead signals with and without adjustment for potential covariates

|  |  |  | **Discovery^c^** | | | | **Replication^d^** | | | **Combined** | | | | |
| --- | --- | --- | --- | --- | --- | --- | --- | --- | --- | --- | --- | --- | --- | --- |
| **Adjustment of the SPC for** | **M** | **SNP^b^** | **N** | **β(SE)** | **p** | **N** | | **β(SE)** | **p** | | **N** | **β(SE)** | **p** | **Het p** |
| Internalising problems^a^ | unadj | rs9257616_G | 5530 | 0.086(0.018) | 3.23E-06 | 5530 | | 0.17(0.082) | 0.082 | | 1131 | 0.089(0.018) | 6.0E-07 | 0.33 |
|  | adj |  | 5530 | 0.081(0.018) | 7.27E-06 | 5530 | | 0.15(0.080) | 0.080 | | 1131 | 0.085(0.018) | 1.7E-06 | 0.42 |
| Hearing problems^a^ | unadj | rs2352908_G | 4711 | 0.098(0.027) | 3.7E-04 | 1364 | | 0.24(0.10) | 0.023 | | 6075 | 0.11(0.027) | 5.6E-05 | 0.20 |
|  | adj |  | 4711 | 0.097(0.027) | 4.2E-04 | 1364 | | 0.23(0.10) | 0.025 | | 6075 | 0.11(0.027) | 6.8E-05 | 0.21 |

a – In addition adjusted for age and sex and two principal components

b – Coded with respect to the risk allele

c – In ALSPAC, mother-reported internalising problems in children were assessed at 10 years of age using the Strengths-and-Difficulties Questionnaire (SDQ)[8] and dichotomised into high and low scorers according to the recommended banding [8]; Hearing thresholds in children for conventional frequencies were measured using air and bone conduction (GSI 61 clinical audiometer and TDH50P headphones) and classified into hearing problems (Mild or moderate uni- or bilateral hearing impairment ) versus bilateral normal hearing

d – In RAINE, mother-reported internalising problems in children were assessed at 10 years of age using the SDQ[8] and dichotomised into high and low scorers according to the recommended banding [8]; Hearing problems in children were based on parent report at 8 years of age and assessed with the question (‘Ever been diagnosed with a hearing problem’)

Regression estimates (SPC) were obtained using Quasi-Poisson regression and restricted to a data set with complete covariate data. Het p – Heterogeneity *p*-value; M – Regression model, unadj – without adjustment; adj – with adjustment; SPC – Short pragmatic composite score

**References**

1. Ma D, Salyakina D, Jaworski JM, Konidari I, Whitehead PL, Andersen AN, Hoffman JD, Slifer SH, Hedges DJ, Cukier HN, Griswold AJ, McCauley JL, Beecham GW, Wright HH, Abramson RK, Martin ER, Hussman JP, Gilbert JR, Cuccaro ML, Haines JL, Pericak-Vance MA: **A Genome-wide Association Study of Autism Reveals a Common Novel Risk Locus at 5p14.1**. *Ann Human Genet* 2009, **73**:263–273.

2. Wang K, Zhang H, Ma D, Bucan M, Glessner JT, Abrahams BS, Salyakina D, Imielinski M, Bradfield JP, Sleiman PMA, Kim CE, Hou C, Frackelton E, Chiavacci R, Takahashi N, Sakurai T, Rappaport E, Lajonchere CM, Munson J, Estes A, Korvatska O, Piven J, Sonnenblick LI, Alvarez Retuerto AI, Herman EI, Dong H, Hutman T, Sigman M, Ozonoff S, Klin A, et al.: **Common genetic variants on 5p14.1 associate with autism spectrum disorders**. *Nature* 2009, **459**:528–33.

3. Weiss LA, Arking DE, Daly MJ, Chakravarti A: **A genome-wide linkage and association scan reveals novel loci for autism**. *Nature* 2009, **461**:802–808.

4. Salyakina D, Ma DQ, Jaworski JM, Konidari I, Whitehead PL, Henson R, Martinez D, Robinson JL, Sacharow S, Wright HH, Abramson RK, Gilbert JR, Cuccaro ML, Pericak-Vance MA: **Variants in several genomic regions associated with asperger disorder**. *Autism Res* 2010, **3**:303–310.

5. Anney R, Klei L, Pinto D, Regan R, Conroy J, Magalhaes TR, Correia C, Abrahams BS, Sykes N, Pagnamenta AT, Almeida J, Bacchelli E, Bailey AJ, Baird G, Battaglia A, Berney T, Bolshakova N, Bölte S, Bolton PF, Bourgeron T, Brennan S, Brian J, Carson AR, Casallo G, Casey J, Chu SH, Cochrane L, Corsello C, Crawford EL, Crossett A, et al.: **A genome-wide scan for common alleles affecting risk for autism**. *Hum Mol Genet* 2010, **15**:4072–4082.

6. Liu JZ, Mcrae AF, Nyholt DR, Medland SE, Wray NR, Brown KM, Hayward NK, Montgomery GW, Visscher PM, Martin NG, Macgregor S: **A Versatile Gene-Based Test for Genome-wide Association Studies**. *Am J Hum Genet* 2010, **87**:139–145.

7. **RegulomeDB** [http://regulome.stanford.edu/]

8. Goodman R: **The Strengths and Difficulties Questionnaire: a research note**. *J Child Psychol Psychiatry* 1997, **38**:581–586.

9. Wechsler D, Golombok J, Rust J: *WISC-IIIUK Wechsler Intelligence Scale for Children – UK Manual*. 3rd edition. Sidcup, UK: The Psychological Corporation; 1992.

10. Dunn L, Dunn L: *Peabody Picture Vocabulary Test-Revised: Manual*. MN: American Guidance Services: Circle Pines; 1981.

11. **LocusZoom - Create Plots of Genetic Data** [http://csg.sph.umich.edu/locuszoom/]

**Additional Figures**

**Figure S1**: Histogram of the short pragmatic composite score (SPC) in ALSPAC before reverse-coding.

**
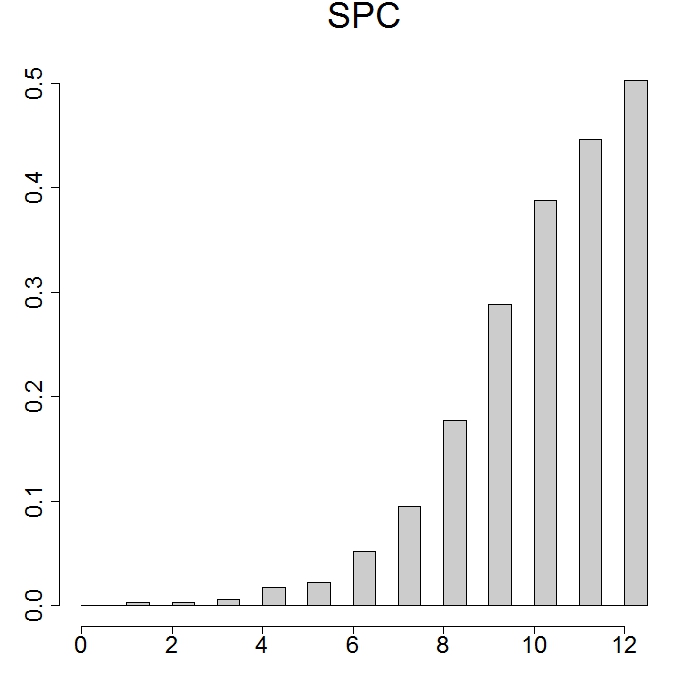
**

**Figure S2**: Regional association plot (Build 36) for the top 5 independent regions in the ALSPAC cohort, which did not achieve replication in RAINE, ordered by significance in the discovery analysis. All association plots were generated with the Locuszoom software [11].

**a**

**b**

**c**

**d**

**e**
